# Pathogenicity and virulence of henipaviruses

**DOI:** 10.1080/21505594.2023.2273684

**Published:** 2023-11-10

**Authors:** Benjamin Kaza, Hector C. Aguilar

**Affiliations:** a Department of Microbiology, College of Agriculture and Life Sciences, Cornell University, Ithaca, NY, USA; b Department of Microbiology and Immunology, College of Veterinary Medicine, Cornell University

**Keywords:** Virus, Virulence, Henipavirus, Paramyxovirus, Pathogenesis

## Abstract

Paramyxoviruses are a family of single-stranded negative-sense RNA viruses, many of which are responsible for a range of respiratory and neurological diseases in humans and animals. Among the most notable are the henipaviruses, which include the deadly Nipah (NiV) and Hendra (HeV) viruses, the causative agents of outbreaks of severe disease and high case fatality rates in humans and animals. NiV and HeV are maintained in fruit bat reservoirs primarily in the family *Pteropus* and spillover into humans directly or by an intermediate amplifying host such as swine or horses. Recently, non-chiropteran associated Langya (LayV), Gamak (GAKV), and Mojiang (MojV) viruses have been discovered with confirmed or suspected ability to cause disease in humans or animals. These viruses are less genetically related to HeV and NiV yet share many features with their better-known counterparts. Recent advances in surveillance of wild animal reservoir viruses have revealed a high number of henipaviral genome sequences distributed across most continents, and mammalian orders previously unknown to harbour henipaviruses. In this review, we summarize the current knowledge on the range of pathogenesis observed for the henipaviruses as well as their replication cycle, epidemiology, genomics, and host responses. We focus on the most pathogenic viruses, including NiV, HeV, LayV, and GAKV, as well as the experimentally non-pathogenic CedV. We also highlight the emerging threats posed by these and potentially other closely related viruses.

## Introduction

Paramyxoviruses are a diverse group of negative-sense non-segmented RNA viruses that are responsible for a range of respiratory and neurological diseases in humans and animals [1]. Among the most notable of these viruses are the henipaviruses: Nipah (NiV) and Hendra (HeV) viruses, which have caused several outbreaks of severe disease in humans and horses in Southeast Asia and Australia [2]. In recent years, two novel genetically related viruses, Langya (LayV), and Mojiang (MojV) viruses, emerged in China and have been found to infect shrews and rats, respectively. These viruses have either shown the ability or the potential to cause disease in humans, respectively [3,4]. In this review, we provide a comprehensive overview of the current knowledge on the pathogenesis, life cycle, epidemiology, genomics, and host responses of these and closely related emerging henipaviruses.

**Figure 1.**
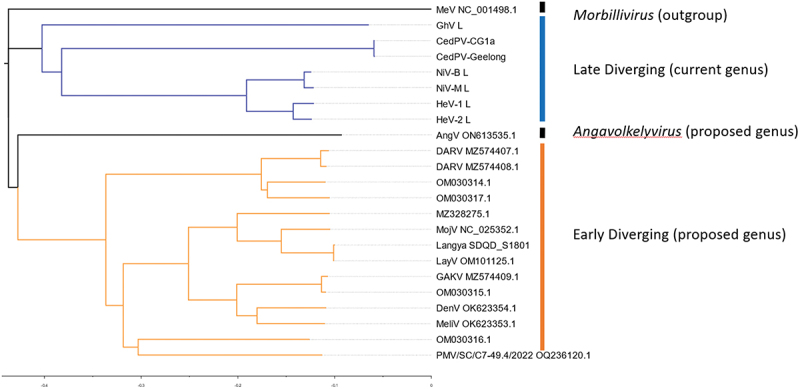
Neighbor end joining tree of L protein CDS for selected *paramyxoviruses*. L CDS neighbor end joining homology tree made with Tamara Nei consensus sequence of 23 sequences. L CDS from MeV (NC_001498.1), GhV (HQ660129), CedPV-CG1a (NC_025351), CedPV-Geelong (KP271122), NiV-B (AY988601), NiV-M (NC_002728), HeV (AF017149), HeV (MZ318101), AngV (ON613535), DARV (MZ574408), MojV, (NC_025352), LayV (OM101125), GAKV (MZ574409), DenV (OK623355), and MeliV (OK623354).

**Figure 2.**
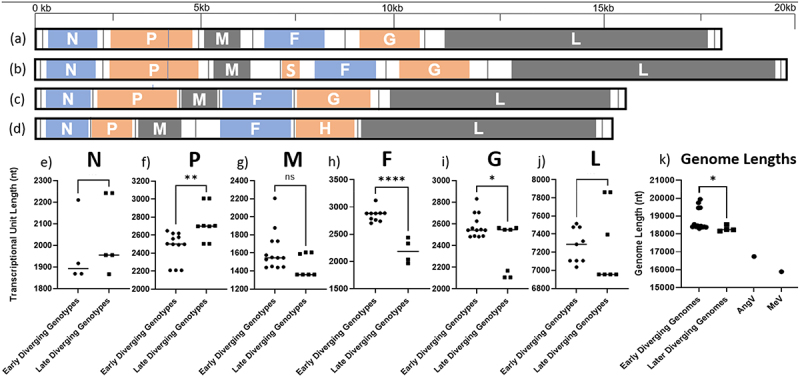
Genomic organization of *henipaviruses* and related species: of the selected genomes (a) the HeV genome has the conserved gene order N-P-M-F-G-L where each ORF is encoded on a separate mRNA which differs from (b) which is the MeliV genome characteristic of LayV, MojV, GAKV, DARV, and related viruses where the F gene encodes an additional ORF X in the 5’ UTR producing the S protein. AngV (c) has the smallest genome and each mRNA is likely monocistronic. MeV (d) which is representative of the morbillivirus genus as an outgroup is markedly smaller compared to (a) and (b). The transcriptional unit length (all nucleotides from last intergenic residue to the first residue of the next intergenic region) from each major branch of the henipavirus phylogeny compared to each other for (e) the N gene of genomes with intergenic region present in the leader, (f) for P gene in all genomes, (g) M in all genomes, (h) F transcriptional unit (including ORF X/S protein CDS for early diverging genomes), (i) G gene, (j) for the L gene in all genomes with intergenic sequence in the trailer, and (k) total reported genome length. The length of early diverging henipavirus genomes (including LayV and MojV) is longer compared to later diverging genomes (including HeV, NiV, and CedV) and the transcriptional unit encoding the F protein and/or S protein is significantly longer (f).

In addition to mounting serological evidence that henipaviruses have a broad geographical and host species distribution, since the initial characterization of HeV in 1994 over 14 genotypes have been deposited to Genbank clustering between HeV and MojV [5–10] (Table 1). The MojV genome, *Henipavirus mojiangense* Tongguan 1, is highly divergent from the prototypic HeV genome, *Henipavirus hendraense* Geelong, but was officially included in the henipavirus genus by the International Committee on Taxonomy of Viruses (ICTV), which has broadened the inclusion basis for the *Henipavirus* genus. As an effect of this inclusion, there are highly diverse genomes deposited on Genbank that have been found on 5 different continents, primarily from shrews. Of these new genomes, only a few viruses have been isolated, including LayV [3,5,11] (Table 2). Most of the viruses are more like MojV or LayV viruses than the prototypic HeV genome, indicating that these viruses may eventually be considered in a separate genus in combination with observed differences in terms of phylogenetic distance [5,11] (Figure 1). Additionally, there are two viruses that are roughly equally less related to HeV as they are to LayV or MojV viruses: Peixe-Boi virus (PBV, a partial genome sequence detected from a Brazilian possum) and Angalovokely virus (AngV) [6,9]. These sequences are highly divergent from the prototypic henipaviruses and arguably may not fulfill the International Committee for the Taxonomy of Viruses (ICTV) inclusion criteria for this genus. AngV and PBV have not been associated with any human disease to date and are not expected to bind ephrin receptors, at least in the case of AngV [6,9]. The genomic structure and organization of the newly included viruses is also a striking difference (Figure 2), which has been proposed as a distinguishing feature of proposed taxa within the current *Henipavirus* genus [11].

**Table 1.** Annotated genomes related to HeV and NiV. list of nearly or completely sequenced genomes related to HeV or NiV used in this review and their corresponding GenBank accession numbers.

Virus Name	Accession Number
Hendra virus isolate Geelong (genotype 1)	AF017149
Nipah virus (Malaysian strain)	NC_002728
Nipah virus (Bangladesh strain)	AY988601
Ghanaian bat henipavirus	HQ660129
Cedar virus	JQ001776
Mojiang virus isolate Tongguan1	NC_025352
Hendra virus (genotype 2)	MZ318101
Wenzhou Apodemus agrarius henipavirus 1	MZ328275
Daeryong virus Cs17–46	MZ574408
Daeryong virus Cl17–32	MZ574407
Gamak virus Cs17–65	MZ574409
Jingmen Crocidura shantungensis henipavirus 1 isolate SYS_SheQu	OM030314
Jingmen Crocidura shantungensis henipavirus 2 isolate SYS_SheQu	OM030315
Wufeng Chodsigoa smithii henipavirus 1 isolate WFS_ChangWei	OM030316
Wufeng Crocidura attenuata henipavirus 1 isolate WFS_SheQu	OM030317
Melian virus	OK623354
Denwin virus	OK623355
Langya virus	OM101125
Angavokely henipavirus	ON613535
Peixe-Boi virus	MZ615319
Chodsigoa hypsibia henipavirus isolate PMV/SC/C7–49.4/2022	OQ236120
Crocidura tanakae henipavirus isolate Shiyan201	OQ970176

**Table 2.** Current virus isolates. Currently HeV, NiV, and CedV are the only viruses for which there is a live virus isolate and reverse genetics system available. GAKV and LayV have been isolated and shown to replicate in human cells [3,12].

Virus Isolate	Date Isolated	CFR% in Humans	Country of Isolation	Animals Species Susceptible	Reverse Genetic System
NiV	1998	40–70%	Peninsular Malaysia, India, Bangladesh, Philippines (presumed)	Humans, bats, pigs, hamsters, guinea pigs, ferrets, mice, horses (presumed)	Yes
HeV	1994	57% (4/7)	Australia	Humans, horses, bats, hamsters, mice, dogs, cats, ferrets	Yes
CedPV	2012	-	Australia	Bats,	Yes
GAKV	2021	-	South Korea	Shrews, human cells	No
LayV	2022	0% (0/35)	China	Humans, shrews	No

## Epidemiology

**Figure 3.**
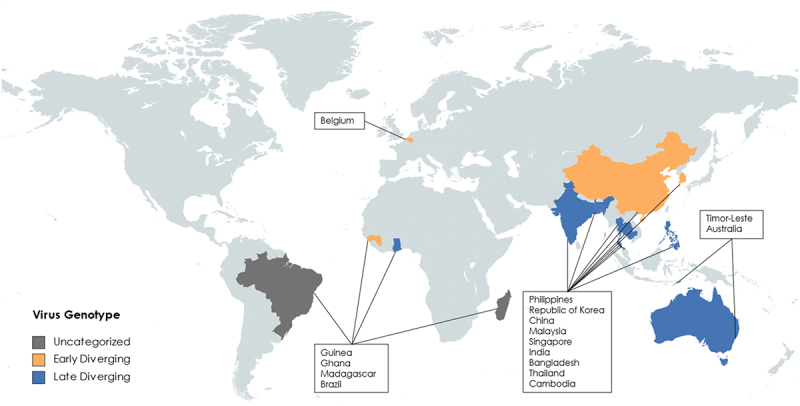
Map of where henipavirus related genomes have been sequenced or detected. Countries are subdivided into colours by proposed genotype, unchategorized genotypes are grey, early diverging genotypes are in Orange, late diverging genotypes are in blue. (created with mapchart.net).

The first henipavirus discovered, HeV, was first identified in Western Australia in 1994 [12]. The initial outbreak affected 21 horses with severe respiratory infections in Hendra, Brisbane (Figure 3). During this outbreak, two humans were also infected with HeV while caring for sick horses. The human patients experienced flu-like symptoms, one of which developed further respiratory and cardiopulmonary signs before dying of cardiovascular complications from an arterial thrombosis in his leg. Of the 7 recorded HeV human cases, the incubation period has been estimated to be 9–16 days. Since these initial infections, there have been 83 laboratory confirmed cases in horses along the Queensland coast with an 89% case fatality rate (CFR) and seven human cases with a 57% CFR [13–17]. Fruit bats of the *Pteropus* genus were identified as the natural reservoirs of HeV, and it is believed that horses act as intermediate amplifying hosts after being infected by eating partially eaten fruit or through contact with infected bat urine [18,19].

NiV emerged during a 1998–1999 epizootic outbreak in peninsular Malaysia and Singapore [20] NiV was first isolated from a case in southwestern Malaysia in Sungai, Nipah [21] (Figure 3). The disease was first recognized as a dual outbreak in pigs and pig farmers/abattoir workers [22]. Pigs developed a mild febrile illness with respiratory involvement, while humans developed severe encephalitic illness. The incubation period in humans was between 4 days to 2 months, with most cases between 6 and 9 days, and the onset was abrupt, with fever, headache, dizziness, and vomiting. Later neurological signs included reduced levels of consciousness, areflexia, hypotonia, and abnormal doll’s eye-reflex. Nearly a quarter of patients had seizures, and almost all seizures were generalized tonic clonic. In Malaysia, focal neurological signs were common, with the most prominent being segmental myoclonus in 32% of cases [22–25]. The Malaysian NiV outbreak resulted in 265 human cases with a 38% CFR and the culling of over one million pigs. Again, *Pteropus* bats were found to be the reservoir of infection, and it is speculated that partially eaten fruits first infected pigs in farms adjacent to the natural rainforest habitat of the *Pteropus* bats [23]. No other outbreaks have been detected in Malaysia or Singapore.

There was a suspected NiV outbreak in the Philippines (Figure 3) in 2014, associated with transmission from infected horses to humans, which appears to have been caused by the NiV-Malaysia (NiV-M) or a closely related strain, based on a partial genome sequence of the L gene obtained retrospectively [26]. In this outbreak several mammalian species were thought to be infected, including horses, dogs, and humans.

Since 2001 there have been nearly yearly outbreaks of NiV infection in Bangladesh and India (Figure 3), associated with approximately 300 human cases, person-to-person transmission, and a ~ 75% CFR [27–29]. Most of these cases have occurred in Bangladesh or Western Bengal, India, but in 2018 there was an outbreak in Kerala on the Western coast of India, demonstrating the widespread distribution of this virus across the Indian subcontinent [30]. The primary mode of transmission is through direct contact with infected bats, pigs, or other humans. In some cases, transmission has also occurred through consumption of contaminated food or drink, which is thought to be a primary risk factor [31]. The disease is caused by the Bangladesh strain of Nipah virus, (NiV-B), which causes pathology ranging from asymptomatic to severe respiratory illness and neurological disease. While NiV-B is 91.8% similar to NiV-M at the nucleotide level over the entire genome and 91% to 98% at the protein level (based on MUSCLE alignment), outbreaks caused by NiV-B have been associated with higher CFR, more extensive infection in the respiratory tract, and a greater incidence of human-to-human transmission [21,26]. In addition to differences in pathogenesis and geographical distribution, the NiV-B strain also appears to have a shorter incubation period (~9 days) than the originally characterized NiV-M strain [32].

In 2012, a new henipavirus named *Henipavirus cedarense* (CedV) was described by researchers in Australia (Figure 3) through an ongoing surveillance program of paramyxoviruses in Australian fruit bats [33]. Although they were admittedly attempting to detect HeV variants, they detected the genome of the more distantly related CedV from pooled *Pteropus alecto* urine. CedV is currently the most genetically related virus to HeV and NiV and was initially observed to be antigenically cross-reactive with HeV by indirect immunofluorescence of CedV and HeV infected cells. In contrast to the highly pathogenic HeV and NiV, CedV is apathogenic in guinea pig, ferret, and hamster models. In hamsters it is thought that CedV fails to suppress the innate immune system, which leads to the animal being able to restrict CedV replication and clear the infection earlier than for NiV [34]. This difference is likely attributable to the fact that CedV is the only paramyxovirus known to lack the RNA editing site necessary to produce the paramyxoviral accessory proteins V and W, which are known to antagonize the innate immune system by sequestering STAT1 and STAT2. By lacking V and W, CedV is susceptible to the interferon-induced effects of the cellular innate immune system and the infection cycle is arrested.

In June 2012, six miners from Mòjiāng Hani Autonomous County in China (Figure 3) contracted a severe form of pneumonia and three of the miners subsequently died [4]. It is believed that they contracted the disease while working in a cave. Upon investigation of the cave, bat, rat, and shrew samples were collected. These samples led to the discovery of a rat (*Rattus flavipectus*) paramyxoviral genome known as Mòjiāng virus (MojV). The MojV genome was found to be most closely related to viruses within the *Henipavirus* genus and was subsequently classified as a species of *Henipavirus* by the ICTV. Notably, MojV was the most divergent member of the genus to be included at the time and was not found to bind the receptors (ephrins) known to be used by the other members of the genus [4,35,36]. Little is known about MojV because no virus was isolated when it was discovered, thus there is no live virus available for use in animal models. MojV was the first henipavirus described to not bind the canonical ephrin-B2/-B3 receptors. Molecular studies have failed to identify any ephrin receptor as the cellular entry receptor and no surrogate model has been developed to reliably investigate cell entry, replication, pathogenesis, or the host immune response [35,36]. For almost a decade after the genome was published, no henipavirus was isolated that was more genetically related to MojV than either HeV or NiV, which hindered the use of a similar virus to make associative predictions about MojV.

In 2022, a NEJM article reported the discovery of a new virus named *Langya virus* (LayV), in a throat swab sample from a patient during surveillance of febrile patients in eastern China (Figure 3). The initial symptom onset for the first patient was dated December 2018. A subsequent investigation revealed 35 patients with LayV infection, among whom 26 were infected with LayV only (ie. LayV was the only pathogen diagnosed), giving high confidence, yet not definitive proof, that LayV was the aetiological cause of febrile illness. Patients with acute LayV infection presented with symptoms such as fever, fatigue, cough, anorexia, myalgia, nausea, headache, vomiting, thrombocytopenia, leucopenia, and impaired liver and kidney function. LayV was also detected in shrews, suggesting that LayV may be zoonotic, and that shrews may be a natural viral reservoir. Although human-to-human transmission has been reported for other henipaviruses infecting humans, there was no obvious spatial or temporal aggregation of human cases or the assigned haplotypes based on three common single-nucleotide polymorphisms. Interestingly, contact tracing revealed no close-contact LayV transmission [3].

## Genomics

NiV, HeV, and MojV are officially classified within the genus *Henipavirus* by the ICTV. LayV also fits the current inclusion criteria but has not yet been officially included. LayV and MojV are less related to NiV and HeV but share many important features with NiV and HeV that are predictive of their possible pathogenesis, tissue tropism, and ability to evade the immune system of their respective host. NiV, HeV, MojV, and LayV genomes all encode the canonical paramyxoviral genes: N, P, and L for replication, transcription, and packaging of their genome- and structural genes M, F, and G for formation and packaging of the virion, entry/egress, and fusion of host cells to form syncytia [6,9].

**Figure 4.**
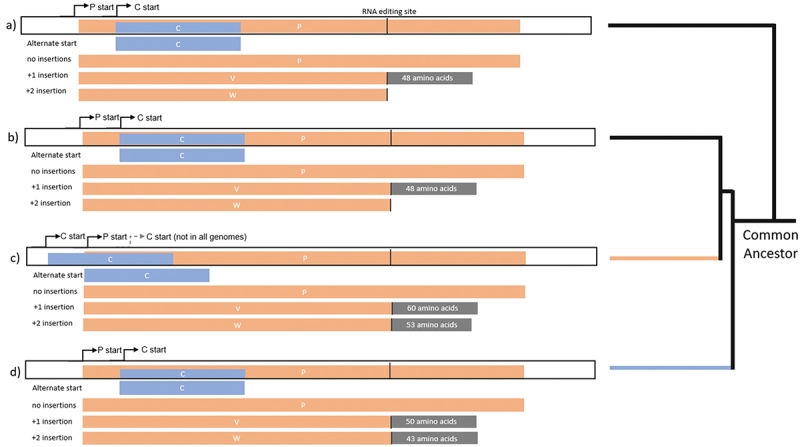
Comparisons of P genes from selected genotypes (a) MeV, (b) AngV, (c) LayV, MojV, GAKV, (d) NiV, HeV, GhV.

All known paramyxoviruses, except for CedV, encode an RNA editing sequence that enables the RNA-dependent RNA-polymerase (L) to execute preprogrammed frameshifts that result in essentially truncated versions of the P protein called V and W. Both V and W contain zinc-finger binding domains and an internally disordered C-terminal end that results from the programmed frameshift relative to the normal P coding sequence [37–40]. The C terminal end of the W protein has a nuclear localization signal that results in W being trafficked to the nucleus. An alternative start codon within the P mRNA results in translation of protein C (Figure 4) [37,38].

In some species the C protein start codon is upstream of the P start codon, while in other species the C start codon is downstream of the P start. In the later diverging henipavirus species, HeV, NiV, CedV, and GhV, the C protein start codon is always internal to the P ORF. However, in more than half of the early-diverging henipaviruses, including MojV, MeliV, DenV, LayV, and GAKV, the C start codon appears to precede the P start codon (Figure 4). The presence of an upstream C start codon seems to follow phylogenetic boundaries within the early-diverging henipaviruses and appears to be more recently evolved. Of note, in the original annotation of the MojV Tongguan 1 genome published to Genbank, the C protein is annotated with an internal start codon [4]. However, this gene may have an unannotated N-terminal extension given a different start codon preceding the P protein start (Figure 4). Given the presence of a similar start position for C in closely related viruses, it appears likely that the MojV Tongguan1 genome was misannotated [11,41].

In the case of NiV, the NiV-P gene encodes two additional proteins V and W through non-templated guanosine insertions at an RNA editing site (+1 and + 2, respectively) by the RNA-dependent RNA-polymerase (L) on the P gene mRNA [40,42,43]. This results in C-terminal frameshifting relative to the P ORF, whereby V and W proteins share the same N termini as the P protein and have shorter, completely different C-terminal ends. In the case of W, the frameshifted C-terminal end encodes a nuclear localization signal (NLS) that facilitates its transport to the nucleus where it interacts with the activated form of IRF3. The V protein is predominantly found in the cytoplasm of infected cells where it interferes with the cellular RNA sensor MDA-5 [42,43]. Interestingly, CedV seems to have lost the functionality of the RNA editing site required to direct the polymerase to add non-templated guanosine residues and reportedly is incapable of producing V or W proteins, which differs from all other known paramyxoviruses [33].

One of the hallmarks of NiV and HeV relative to other paramyxoviruses is the C-terminal extension of the W protein beyond the RNA editing site. In morbilliviruses and respiroviruses, the W protein is essentially the N-terminal portion of the P protein and ends very close to the RNA editing site. The fact that the W protein of AngV does not extend beyond the editing site (Figure 4). This is another genomic feature that separates AngV from the other bat-borne henipaviruses NiV, HeV, and GhV and even the other early diverging viruses. Given that the lack of V and W protein functionality is largely attributable to the absence of pathogenesis observed for CedV in experimentally infected animals, it is reasonable to speculate that the lack of an AngV W C-terminal sequence encoding an NLS may render AngV less pathogenic in at least some animal models, due to AngV W not being able to localize correctly to suppress activated IRF3 [37,42,43].

**Figure 5.**
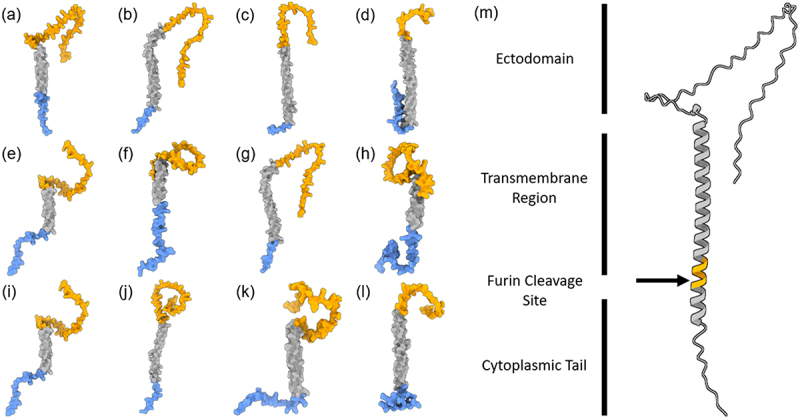
Predicted structures of S proteins. highest scoring Alphafold predictions with regions in blue: N-terminal and predicted intracellular region, Grey: predicted transmembrane region, Orange: predicted ectodomain of S protein CDS from (a) LayV SDQD_H1801 [OM101125], (b) Jingmen Crocidura shantungensis henipavirus 2 isolate SYS_SheQu [OM030315], (c) DewiV strain BE/Ninove/Cr/1/2019 [OK623354], (d) Melian virus strain GN/Meliandou/Cg/1/2018 [OK623353], (e) GAKV Cs17-65 [MZ574409], (f) Wufeng Crocidura attenuata henipavirus 1 isolate WFS_SheQu [OM030317], (g) MojV isolate Tongguan1 [NC_025352], (h) DARV Cl17-46 [MZ574408], (i) GAKV Cs17-65 [MZ574409], (j) MAG: chodsigoa hypsibia henipavirus isolate PMV/SC/C7-49.4/2022 [OQ236120], (k) Wenzhou apodemus agrarius henipavirus 1 [MZ328275], (l) Wufeng Chodsigoa smithii henipavirus 1 isolate WFS_ChangWei [OM030316], (m) schematic of LayV S protein [OM101125] with furin cleavage site in orange.

Interestingly, early diverging genomes from the classical bat-borne henipaviruses contain an additional ORF located in the 5’ UTR of the F gene encoding a predicted small transmembrane protein (Figure 2). Vanmechelen et al. [11] proposed that the presence of an “ORF X” encoding for a transmembrane protein is a hallmark of a separate clade within the henipaviruses, including Melian and Denwin viruses [11]. Given that there is currently no name for the protein encoded by the putative ORF X, we will refer to the small transmembrane (S) protein in this review (Figure 5). To date, no study has investigated the function of the S protein or the apparent bicistronic translation mechanism for the mRNAs encoded by early diverging henipaviruses. Paramyxoviruses typically encode monocistronic mRNAs where translation is cap dependent [44]. In the case of the S protein, translation is likely still driven by ribosomes that are recruited to the 5’ cap. However, the question remains how the translation of the F protein is likely disrupted due to the presence of an upstream ORF (uORF). IRES elements have never been reported in a negative sense virus, so it is likely that some other mechanism enables the dual translation of the S and F proteins from a single mRNA. A comparison of transcriptional unit length from genomes when divided by S-gene encoding “early-diverging” and “late diverging” genotypes indicates that the F gene, encoding both S and F proteins, is significantly longer (*p* = <0.001) in the early diverging genotypes and may explain the longer than expected genomes of the clade (Figure 2). There isn’t a clear correlation between paramyxoviral genome length and the ability to cause human disease. However, shorter henipaviral genomes seem to infect bat hosts regardless of their phylogenetic relatedness, whereas longer, S protein encoding viruses, were only identified from fossorial mammals and particularly from shrews. Of note, no S protein encoding virus has been identified from bats, suggesting that the S protein may function in non-chiropteran hosts. LayV is capable of infecting humans, so the presence of the S protein likely does not restrict transmission from the reservoir host to humans. Interestingly, LayV is the only S-encoding virus with a Furin cleavage site encoded near the N-terminus of the protein (Figure 5). Peixe-Boi virus is the only virus detected from a Possum host but the total genome length for this putative species is unknown. The genetic diversity of these viruses suggests that they may be more widespread than previously thought and may use potentially overlooked genetic mechanisms.

The discovery of new henipavirus-like sequences and the compilation of near-whole genome sequences for almost every proposed species-level genotype has demonstrated that there are likely sub-clades within the previously proposed *Henipavirus* genus (officially containing MojV, NiV, HeV, GhV and CedV) (Figure 3). Arguably, the clade including LayV, MojV, GAKV, DARV, DenWV, and MeliV may be considered a separate genus in the future given its phylogenetic distance and exceptional genomic organization and length. The position of AngV is unclear given its early branch point and potentially incomplete genome sequence. However, if genomic organization alone is considered, AngV may constitute an equally separate genus-level taxonomic distinction (Figure 2). It is likely that the phylogenetic position of AngV will become clear when there are more complete early-diverging sequences available to resolve earlier branch points due to better support of branches early in the tree (Figure 3).

## Molecular virology

Henipaviruses are inherently difficult to study because 1. few live viruses or full viral genomes have been isolated (Tables 1 and 2). NiV and HeV are limited to studies in high containment laboratories (primarily BSL4) and regulated by biosecurity restrictions [45]. As a result, studies on the molecular mechanisms of pathogenesis outside of high-level containment facilities is challenging [21]. Despite these practical constraints, molecular virology methods and bioinformatics have yielded substantial insights into the basic virology of these viruses outside of BSL4. Expression of singular viral proteins from plasmid-based expression systems in mammalian cell culture/organoids, the creation of BSL2 pseudotyped viruses that incorporate henipaviral glycoproteins, and the development of reverse genetics systems have enabled researchers to learn more about henipavirus entry, replication, and egress while also informing the development of countermeasures. Recently, the discovery of apathogenic henipaviruses (such as CedV) to study in lower containment settings have provided insights into henipaviral molecular pathogenesis in concert with findings from NiV and HeV infection experiments performed at BSL4 and clinical and laboratory findings from viral outbreaks [18,46–52]. With the increasing diversity and number of relatively less pathogenic henipavirus isolates than NiV or HeV (such as CedV, LayV, and GAKV), researchers will have access to an unprecedented array of genetic models to safely investigate henipaviruses in low containment settings [3,5,6,8,9,11,33,41].

Paramyxoviruses are enveloped viruses that enter host cells via direct fusion at the plasma membrane. The henipaviral receptor-binding protein (designated G for the henipaviruses, H or HN for other paramyxoviruses) binds to cellular receptors, causing a series of conformational changes in G that in turn trigger the fusion protein (F) to fuse the viral envelope with the host cell membrane and release of the viral nucleocapsid into the cytoplasm [53,54]. For NiV and HeV [as well as the apathogenic CedV and the Ghanaian bat henipavirus (GhV)], the cellular receptors are ephrins. For NiV and HeV, ephrin-B2 and ephrin-B3 are the known cellular receptors, although NiV binds both receptors, and particularly ephrin-B3 at higher affinity than HeV [49,55]. Importantly, the locations of the ephrin-B2 receptor in endothelial cells and neurons, and of ephrinB3 in the brain stem are consistent with viral tropism and the sites of pathological disease [55]^6061^, [56]. CedV can bind ephrin-B1, -B2, -B3, and -A5. It is known that MojV cannot use ephrin receptors, however there is some evidence that ephrin-A4 is an attachment factor for MojV-G, with some variation according to whether it is mouse or human ephrin-A4 [36]. The viral nucleocapsid contains the viral RNA genome, which is transcribed and replicated by the viral polymerase complex consisting of viral proteins N, P, and L. The newly synthesized viral proteins and RNA are assembled into new virus particles, which are assembled at and budded from the infected host cell plasma membrane.

## Pathogenesis

A pathognomonic feature of henipaviruses is cell-cell fusion of neighbouring cells, allowing the virus to spread from infected to naïve cells without budding. The replication cycle of LayV and MojV viruses is not yet known because MojV was never isolated and LayV was isolated very recently. LayV has been shown to replicate in Vero cells in vitro [3]. MojV has been non-causatively associated with pneumonia in humans [4,57]. LayV has been found to cause a febrile respiratory disease in humans with no encephalitis or neurological manifestations [3]. The symptoms of LayV infection are similar to those of other respiratory viruses, such as influenza and SARS-CoV-2, potentially making it difficult to diagnose based on clinical presentation alone (Table 3). MojV has been controversially associated with pneumonia in humans, although it has not been definitively linked to human disease.

**Table 3.** Pathogenic differences between henipavirus outbreaks. symptoms, signs, and laboratory findings associated with outbreaks caused by Nipah virus Malaysia strain, Hendra virus genotype 1, Langya virus H1801, and an outbreak of unknown aetiology that has been speculated to be associated with Mojiang virus Tongguan1.

Symptoms/Signs	Nipah-M [97]	Hendra [7]	Langya [35]	Mojiang? [6]
Fever	97%		100%	100%
Headache	65%		35%	50%
Cough	13%		50%	100%
Neurological Signs	11%	50%	0	0
Vomiting	27%		35%	16%
Elevated Neutrophils			50%	
Thrombocytopenia	30%		57%	
Leukopenia	11%		54%	33%
Death	32%	57%	0	50%

### NiV and HeV pathogenesis

NiV and HeV are neurotropic and can cause severe respiratory and neurological symptoms in humans and animals [21,58–63]. The pathogenesis of these viruses is complex and involves multiple cellular and molecular mechanisms. Upon entry into host cells, NiV and HeV initiate the transcription and replication of their RNA genome. These viruses have developed several strategies to evade and suppress the host innate immune response, including inhibition of type I interferon production and signalling (including IFNA7, RIG-I, and MDA-5), degradation of cellular antiviral factors (including TRIM6), and inhibition of apoptosis [34,39,64–69]. As a result, infected cells can continue to produce viral particles, which spread to neighbouring cells via budding and infection of naïve cells, or viral genomes can spread to naïve cells via cell–cell fusion, causing tissue damage. The respiratory tract is a primary site of NiV and HeV infection in humans, and infected respiratory epithelial cells can produce a large amount of virus particles, leading to severe respiratory symptoms and potentially life-threatening acute respiratory distress syndrome (ARDS) [20,22,24,70–76].

In addition to the respiratory tract, NiV and HeV can also infect the central nervous system (CNS), where they can cause encephalitis, characterized by seizures, confusion, and loss of consciousness [22,27,30,73,75,76]. The mechanisms underlying CNS pathogenesis are not yet fully understood, but it is thought that viral replication in neurons and the vasculature of the brain, as well as immune-mediated damage, play a role [56,77–79]. The early production of type I interferons and other cytokines by infected cells can activate antiviral defences and recruit immune cells to the site of infection [39,40,77]. However, these viruses have developed mechanisms to evade or suppress innate immune responses, allowing for persistent viral replication and the development of severe disease [39,66–68]. The adaptive immune response to NiV and HeV is thought to be important for controlling viral replication and preventing reinfection [73–75,80,81]. Neutralizing antibodies against the viral receptor-binding protein G or the fusion protein F have been shown to be protective in animal models and are thought to be important for controlling viral spread in humans [62,82–84].

HeV and NiV infection cause extensive vasculitis in the lungs, kidneys, heart, and CNS of infected humans and animals [77]. The virus may use different pathways for dissemination, such as bloodstream, cell-to-cell transmission, trans-infection, and CNS spread [73,75]. The primary entry route for the virus is oronasal, and during NiV infection, epithelial cells and type II pneumocytes from the bronchiole are the primary targets in the respiratory tract [73]. NiV targets epithelial, endothelial, and neuronal cells but notably lacks tropism for immune cells [74,75]. The virus induces the production of inflammatory cytokines in infected cells, which can recruit immune cells to the site of infection, subsequently spreading to endothelial cells and gaining entry into the bloodstream [76].

**Figure 6.**
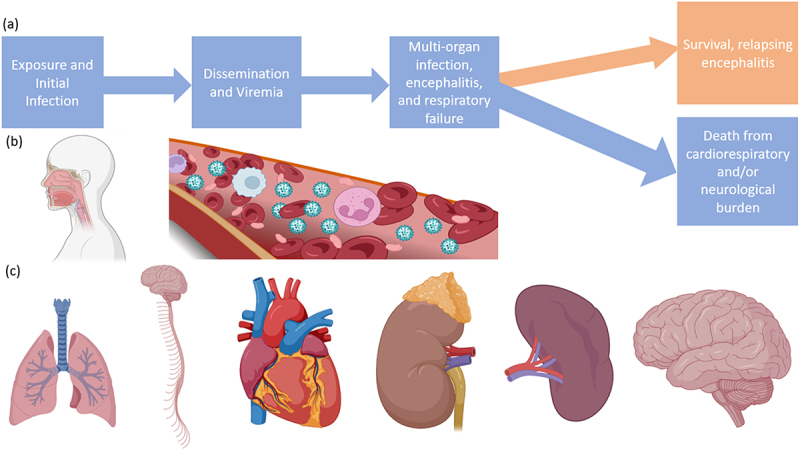
Progression of infection with NiV or HeV. (a) Flow chart of a typical infection, (b) routes of virus entry and dissemination, (c) organ targets including the lungs, CNS, heart, kidneys, spleen, and brain. Made with BioRender.

Once in the bloodstream, the virus disseminates to other tissues, leading to multiple-organ-failure syndrome, including the lungs, spleen, kidneys, and brain (Figure 6) [63]. The virus can enter the CNS either through blood vessels of the brain after global dissemination or potentially earlier by transinfection of the olfactory bulbs during the initial oronasal stage, provided the route of infection was respiratory [73,74]. Neuropathogenesis arises from dual mechanisms of vascular disease and direct parenchymal brain infection, showing viral inclusion bodies and necrosis in both the grey and the white matter after endothelial infection and vasculitis because of platelet activation and thrombi, producing microinfarcts [2,58,85]. While vasculitis in the brain has been suggested as a neuropathogenesis mechanism, the highest NiV antigen loads are associated with infected neurons that are distal to vascular infection sites and were not found near thrombi or necroses [82]. Notably, glia are spared from infection, in parallel with the lack of immune-cell infection observed in other organ systems. Often necrotic neural tissue is observed in direct proximity to uninfected glia [2,82].

Direct cytopathic effects of NiV replication in the microvasculature are accompanied by those mediated by inflammatory cytokines (TNF-α and IL1-β) released by microglia or other surrounding cells, promoting BBB disruption [2,82]. It is not understood if systemic virus dissemination directly leads to CNS infection or if infection of the brain occurs by a different mechanism, as in animal models CNS infection becomes apparent at a similar time viraemia is observed. In non-human primate (NHP) models of NiV infection, clinical features mostly reflect the epidemiological and pathological observations from human cases, however, there are some notable differences [58,60,86,87]. In a NiV-B challenge of African Green Monkeys (AGM), infection was uniformly lethal and resulted in a rapidly fatal disease that mostly began with non-specific neurological symptoms including anorexia and depression between 6 and 9 days post infection (dpi), resulting in death at 9–10 dpi. While this model was uniformly fatal, the laboratory findings early during infection were highly variable, with 50% (2/4) animals developing thrombocytopenia. Additionally, half of the animals (2/4) developed leukocytosis and the other half (2/4) developed lymphopenia. Interestingly, chest radiography prior to 10 dpi did not reveal significant findings for any animal [73].

Infiltrating lymphocytes are present during HeV/NiV encephalitis, but the protective role and/or role as mediators of damage of individual inflammatory cell populations within the brain remains to be determined [1,2,60–62,84,86–89]. One study has suggested that leukocytes originating from early sites of infection, including the lung or digestive tract, can carry the virus to other organ systems including the brain without becoming infected [78]. This study also found that dendritic cells were the only type of human lymphocyte susceptible to NiV infection. In some ways this is similar for the human-infecting paramyxovirus Measles morbillivirus (MeV), which efficiently disseminates throughout the host by infection of migrating lymphoid cells. In the case of NiV and HeV, endothelial/epithelial cells and neurons are much more easily infected, expanding the range of tissues they can infect as compared to MeV, consistent with the characteristic highly fatal multi-organ infection caused by NiV and HeV. Overall, HeV and NiV infection is associated with extensive vascular damage and multi-organ failure, with the virus using different pathways for dissemination and CNS invasion. This leads to severe and extensive encephalic pathology and typically death as compared to other paramyxoviruses and unrelated human respiratory viruses such as SARS-CoV-2, although the initial symptoms may be similar [90,91].

While there have been markedly fewer cases of HeV infection in humans, there appears to be more pathological variation [13,69–75]. Of the two first HeV cases, one ultimately had cardiopulmonary and respiratory lesions that led to death while the other developed a severe recrudescent meningitis with extensive infiltrates in addition to the characteristic respiratory disease [12,13,92]. It is unclear whether HeV has a more variable pathogenic phenotype resulting in a longer-term neurological disease or if these possibilities are related to the low number of human HeV cases. It is worth noting that there is at least one other strain of HeV [93], and that some of these pathogenic phenotypes are thought to vary among NiV genotypes (between NiV-B and NiV-M), so it is feasible that HeV disease variability is based on the genetic distance from NiV strains and their better-characterized disease. Alternatively, differences in pathology observed in human cases of HeV could be related to differences in viral load, given that all human cases are presumed to have acquired HeV from an intermediate amplifying horse [12,18,94–96].

### LayV and MojV pathogenesis

In contrast, LayV has been found to cause a febrile respiratory disease in humans with no encephalic or neurological manifestations, while MojV has been associated with (although never definitely proven to cause) pneumonia in humans. If the outbreak of atypical pneumonia associated with MojV is hypothesized to be aetiologically associated with MojV, then both syndromes associated with MojV and LayV are marked by abnormalities in thrombocyte count, leucopenia, and blood chemistry compared to NiV or HeV virus infection [4,57]. While the receptor tropism for NiV, HeV, and CedV are well characterized in animal models and human infections (in the case of HeV and NiV), the receptor is unknown for MojV and LayV, and no animal models have been established for LayV or any closely related virus (despite the existence of virus isolates for both LayV and GAKV) [3,41].

The closest account to actual evidence of MojV infection are laboratory and clinical findings from a pneumonia outbreak of unknown aetiology that six human patients experienced in 2012 after working in the same cave that MojV was retrospectively sequenced from in a rat swab. Blood chemistry and immune markers indicated that the miners likely had an infectious disease. Three out of the six miners who developed the disease succumbed to infection, a 50% CFR. A 2013 Chinese Master’s thesis published by Li Xu at Kunming Medical University described clinical characteristics and laboratory findings of the patients who contracted pneumonia during this outbreak. The outbreak has also been suggested to be attributable to a pathogenic fungi acquired from the cave, so it is unclear if the patients actually were infected with MojV as opposed to another infectious disease. The 50% CFR (3/6) is a unique feature of the outbreak that is shared with few other human-infecting viruses (including the related NiV and HeV). However, the reported levels of C-reactive protein (6/6) are higher than the observed incidence in experimental NHP infections with NiV-B [73].

While LayV is more causatively linked to disease, the mechanisms that lead to disease are still not fully known due to the absence of extensive histopathological analysis such as those available for NiV/HeV cases, due to NiV/HeV patients frequently succumbing to infection and the availability of subsequent necropsy findings [3]. The current evidence available to evaluate the pathogenesis of LayV relies on laboratory findings and symptomology from nonfatal LayV cases, as well as the low genomic information available from only 6 LayV genomes published [3].

## Immune responses and vaccine development

The immune responses to henipaviruses compared to other paramyxoviruses are not fully understood, but recent studies have shed light on the role of innate and adaptive immunity in controlling viral replication and limiting disease severity in animal and cell culture models, which has informed the design and production of several candidate experimental vaccines against NiV and HeV.

In contrast to the non-pathogenic CedV, NiV and HeV evade the early detection by antagonizing the innate immune system for long enough to spread to critical tissues in the central nervous system and respiratory tract, that render the adaptive immune response ineffective at preventing severe disease and death by the time it is mounted [39,39,40,42,43,66–68,73–76,78]. NiV and HeV have evolved a multifaceted approach to suppressing antiviral signalling in early target cells and also blocking autocrine antiviral signalling. NiV-P was recently shown to sequester STAT1/STAT2 into viral inclusion bodies formed by NiV-N and NiV-P proteins, which results in functional paracrine signalling to the uninfected neighbouring cells while antagonizing autocrine signalling [39]. This contrast to other paramyoviruses that strategically target STAT1 and STAT2 for degradation [38]. NiV-N in particular has also been shown to block STAT1 and STAT2 binding. The NiV-C protein is also strongly associated with the suppression of cellular innate antiviral factors including (IL-1β), IL-8, CXCL2, CXCL3, CXCL6, CCL20, and beta interferon [37]. NiV-M has also been experimentally shown to target TRIM-6 for degradation [69].

As for MeV, for NiV and HeV the presence of neutralizing antibodies (NAbs) is thought to be the primary determinant of survival and recovery in animal models and human cases [97]. The first vaccine, comprised of a soluble version of the HeV G protein (HeV-sG), marketed as equiVax, is licenced for use against the prevention of HeV infection in horses in Australia and was the first and currently only vaccine licenced for use against henipavirus infection [80]. While not licenced, monoclonal antibody (MAb) m102.4, raised against the ephrin-binding site of HeV/NiV G protein, has been used on a compassionate basis as post-exposure prophylaxis in cases of high-risk human exposure to HeV in Australia [80]. Given that potent NAbs are strongly correlated with survival and positive outcomes for HeV and NiV, there are many published candidate experimental vaccines in development that aim to confer protection by inducing strongly NAb responses against the two major surface antigens: F and G.

Of the experimental vaccines candidates against NiV and HeV, many are based on the recombinant replication-competent VSV platform, used to make the approved EBOV vaccine [81,97–99]. This VSV platform has a certain level of safety and high efficacy, and is being tested for other highly pathogenic viruses, including EBOV, MARV, and SARS-CoV-2 [100–102]. Other vaccine candidates are based on a genetically modified VSV platform which is replication-deficient to increase safety, while still being able to incorporate heterologous glycoproteins expressed in transfected cells, which are then transduced with the attenuated VSV lacking its own glycoprotein. This strategy has been used to develop an experimental vaccine candidate that confers complete protection against NiV, HeV, and EBOV in a hamster model and is now being tested in a swine model [103]. Yet another experimental vaccine candidate against NiV has been assessed using a similar mRNA platform as the Moderna SARS-CoV-2 vaccine mRNA-1273 [104].

In the case of NiV and HeV, innate immune responses also play a critical role in controlling viral replication and limiting disease severity [39,67,68]. The early production of type I interferons (IFNs) and other cytokines by infected cells can activate antiviral defences and recruit immune cells to the site of infection. However, these viruses have developed mechanisms to evade or suppress innate immune responses, allowing for persistent viral replication and the development of severe disease, attributed primarily to P gene protein products P, V, W, and C, as described above.

The adaptive immune response against NiV and HeV is thought to be important for controlling viral replication and preventing reinfection [34,105]. NAbs against the viral receptor-binding protein have been shown to be protective in animal models and are thought to be important for controlling viral spread in humans [83,106]. NAbs against the F protein may provide broader immunoprotection against henipaviruses [103]. The immune responses to LayV and MojV are not yet well characterized, but it is thought that innate and adaptive immune responses play similar roles in controlling viral replication and limiting disease severity. Interestingly, LayV and potential MojV infection cases seem to correlate with depletion of white blood cells and platelets [3].

## Antiviral development

No antivirals are currently approved for use against NiV, HeV, MojV, or LayV although several antiviral agents have been used on a compassionate use basis, in open-label efficacy studies during outbreaks, or indicated for emergency use in case of an outbreak.

Two monoclonal antibodies (mAbs), m102.4 and h5B3.1, have shown potent activity against NiV in both ferret and NHP models. m102.4 binds the receptor-binding domain of the HeV or NiV receptor binding G, and can be administered post-exposure with the treatment window extending to the onset of clinical symptoms. The protective window was estimated to be 3 days post exposure for NiV-B and HeV, whereas animals could be protected up to 5 days post exposure in the case of NiV-M. In cases of high-risk exposure, m102.4 has been administered to humans on a compassionate use basis 14 times without significant adverse effects. Given the efficacy of m102.4 in animal models and presumed tolerability in humans when administered in emergency situations, a phase I clinical trial was conducted in humans [83]. Neutralizing levels of m102.4 were detected in the serum 8 days after administration. The most reported side effect was headache. The other antibody, h5B3.1, targets the NiV and HeV fusion protein F, blocking a conformational change necessary for the viral protein to function. Similarly, this antibody was effective at protecting ferrets from NiV and HeV challenge when administered 3- or 5-days post exposure [62,82–84].

Ribavirin, a nucleoside analog used in the treatment of other viral infections, was administered to 140 patients who were infected with NiV during the initial 1998 Malaysian/Singaporean outbreak. Another 54 patients were unable to receive ribavirin treatment (either because they refused or were treated prior to the start of the study). In the cohort who received ribavirin treatment, there was reportedly a 36% reduction in death compared to the untreated cohort [89]. However, a statistically significant correlation between ribavirin treatment and prognosis could not been established in this study. In a 2018 outbreak of NiV-B in the Kerala state of India, a subset of patients received ribavirin post-exposure. There was a 20% reduction in mortality among the treated group compared to patients who did not receive treatment, however, the total number of patients (*n* = 23) meant that a statistically significant correlation could not be established for ribavirin treatment and infection outcome [85]. In the hamster models, ribavirin treatment alone or in combination with chloroquine was not protective against lethal NiV-M or HeV challenge [107].

No other specific antiviral therapy has been administered in human cases or NiV, HeV, or LayV infection. However, several candidate antiviral compounds have demonstrated favourable efficacy and safety profiles in animal models, many of which have indications for use for common viral infections such as SARS-CoV-2. Remdesivir, another nucleoside analog, completely protected AGMs from death against lethal NiV-B challenge when administered 24-h post-exposure and through the duration of the viral incubation period [84]. Two out of four animals developed mild respiratory disease that resolved without other intervention, and viral RNA was detected in the brain of a single animal. Favipiravir (*T*-1075), another nucleoside analog, was shown to protect hamsters from mortality and pathology in a lethal NiV-M challenge when administered from time of infection to 14 days post exposure [108].

## Conclusions

In summary, NiV, HeV, LayV, and MojV are emerging threats that have the potential to cause severe respiratory and neurological disease in humans and animals. These viruses are genetically related and share many features, including their entry mechanisms, replication cycle, and immune evasion strategies, with the exceptions described here. Ongoing surveillance and research are necessary to better understand the epidemiology, pathogenesis, and immune responses to these viruses and to develop effective prevention and treatment strategies. These viruses can cause severe respiratory and neurological disease in humans and animals, some with the highest mortality rates known in humans, and their ability to evade and suppress the host immune response plays a critical role in disease pathogenesis. Ongoing research is necessary to better understand the mechanisms underlying disease and ways to combat it.

In the age of high-throughput next generation sequencing and the accessibility of compiling whole viral genomes sequences from animals, it is likely that the rate of henipavirus discovery (and for paramyxoviruses more broadly) will increase and will allow resolution of early branching nodes in the phylogeny for NiV, HeV, LayV and the other related viruses. Given the increase in genome discovery not associated with disease over the last 10 years for the shrew-borne henipavirus relatives, it may be increasingly prudent to predict human pathogenic potential from sequence alone. The development of reverse genetics systems for newly discovered viruses will be helpful to find correlates of human-pathogenesis in surrogate model systems. Additionally, new sequencing technologies will help complete the existing genome sequences for rare henipaviruses (e.g. MojV or AngV), previously not possible due to low sequence coverage. This is critical to advance reverse genetics, minigenome, and infectious clone studies. The current wealth of available henipavirus genome sequences provides ample opportunities to study well-sequenced and well-conserved coding sequences for structural proteins F, G, and M, known determinants of tropism and pathogenic potential. We now know that viruses like NiV and HeV appear to be ubiquitous and diverse. However, we have also discovered that even closely related viruses to NiV or HeV are unlikely to cause the same disease regardless of which receptors they can bind, as in the case of CedV. It is known that NiV, HeV, and LayV can potentially infect and/or cause disease in a broad range of mammalian taxa- but the circumstances which cause spillover are complex and can’t be modelled based on viral genotype alone.

Bioinformatic identification of a gene encoding the novel paramyxoviral S protein from a previously annotated sequence (MojV isolate Tongguan1) and the subsequent discovery that this ORF is conserved among the shrew-borne henipaviruses is a call to reinvestigate what may have been missed in existing sequencing data. The S protein/ORF X could be described as an ORFan gene because it appears to have been acquired recently in the evolutionary record and doesn’t have any easily identifiable homologues. Future studies could yield useful insights on the function of the S protein itself, which is currently unknown, as well as the translation mechanism that allows two ORFs to be encoded on a viral mRNA that typically has just one. This could be accomplished by expressing a plasmid with different reporter ORFs corresponding to the S protein and F protein respectively. The most useful strategy would be using an authentic virus isolate to attempt to detect both S and F proteins in parallel with detecting the mRNA from the same sample. Once an infectious clone of an early diverging henipavirus has been constructed (such as LayV), it would be extremely insightful to knockout the S protein, either by deletion or removing any necessary start codons, to determine what phenotype is lost, if any.

The recent isolation of viruses that cause a different type of disease than NiV and HeV is a major development that will enable researchers to definitively map virulence factors that can be attributed to the most extreme consequences of henipavirus infection (ie encephalitis and multiorgan failure). Unlike other genera of highly pathogenic viruses, such as *Ebolavirus*, until recently the genus *Henipavirus* only had 3 cultured isolates, one of which did not cause disease (CedV). Excitingly, the expansion of the genus has occurred alongside the growing diversification of models that can be used to characterize the pathogenesis and virulence of new members. Namely, this includes the development of human arterial cultures and immunological assays. Despite the paucity of available medical countermeasures against NiV, there are many vaccines and antivirals in preclinical development and at least one in clinical trials. Many of the vaccine candidates are broadly protective and fast acting, with highly favourable safety and efficacy profiles. Given that many are based on approved vaccine platforms, it seems likely that they may be deployed rapidly in an emergency outbreak scenario.

## Data Availability

Data sharing is not applicable to this article as no new data were created or analysed in this study.
